# A retrospective serological survey of hepatitis B virus infection in Northeast China

**DOI:** 10.1186/s12879-019-4091-3

**Published:** 2019-05-20

**Authors:** Jing Meng, Hongqin Xu, Dongming Sui, Jing Jiang, Jie Li, Yanhang Gao, Junqi Niu

**Affiliations:** 1 Department of Hepatology, First Hospital of Jilin University, Changchun, Jilin China; 2 Department of Asset Management, First Hospital of Jilin University, Changchun, Jilin China; 3 Department of Clinical Epidemiology, First Hospital of Jilin University, Changchun, Jilin China; 4 Department of Microbiology and Infectious Diseases Center, School of Basic Medical Sciences, Peking University Health Science Center, Beijing, China

**Keywords:** Inpatients, HBV prevalence, HBsAg, Chronic hepatitis B

## Abstract

**Background:**

Hepatitis B virus (HBV) infection is a major public health burden in China although it has steadily declined over the last two decades. A valid updated prevalence of HBV infection in China relies on a large sample size. Hence this study aimed to estimate HBV seroprevalence using a large inpatient population in Northeast China.

**Methods:**

We consecutively enrolled 218,627 inpatients aged 1–70 years admitted to the First Hospital of Jilin University from January 2010 through December 2014. HBV serological markers were detected by chemiluminescence immunoassay (CLIA).

**Results:**

Among the 218,627 collected samples, 16,254 (7.43%) were positive for HBsAg and 41.64% of patients were negative for all the HBV markers. The highest HBsAg prevalence was 10.05% in the 41–50 year age group and the lowest were 0.47% in the 1–10 and 2.35% in the 11–20 year age groups, respectively. HBsAg positivity was higher in males compared to females (8.94% vs. 5.80%). An HBsAg positivity of nearly 14% was found in middle-aged males, and positivity was 6.2% in females of childbearing age. One-third of this population only had a single HBsAb marker, which was also detected in 60% of patients aged under 20 years.

**Conclusion:**

Though universal hepatitis B vaccination of infants has significantly reduced HBsAg prevalence in children, the number of most adults who have been infected with HBV remains steady. Extra care and resources should be provided to HBV-infected middle-aged males to stop the progression of chronic hepatitis B, and HBsAg positive females of childbearing age to block vertical HBV transmission.

## Background

Hepatitis B virus (HBV) infection remains a huge public health burden in China [1, 2] despite the success of HBV vaccination. Hepatitis B vaccine was first introduced into China in 1978 [1]. In 1985, China initiated HBV vaccination in a small population with a plasma-derived hepatitis B vaccine [3]. In 1992, the Chinese Ministry of Health recommended that infants be routinely vaccinated against HBV, but recipients were charged for both the vaccine and service [4]. As a result, higher percentages of infants in the more affluent larger cities on the eastern coast of China received HBV vaccination compared to infants inland and in rural areas [5]. In 2002, China integrated HBV vaccination into an expanded national immunization program in which HBV vaccines were free of charge [6]. Furthermore, the service fees for HBV vaccination were waived in 2005 [7]. In 2009, China enacted a new policy that enabled children < 15 years of age who had not been vaccinated to receive free vaccinations [8]. In 2011, China started a new program that combined the prevention of mother-to-child transmissions of HIV, syphilis, and HBV in 1156 counties (representing 44% of pregnant women in China) and then expanded it nationwide to cover all pregnancies in 2015 [9].

Since this universal HBV vaccination strategy was implemented among infants from the early 1990s, HBsAg seroprevalence in China has decreased, particularly among children [10]. In 1992, just before the implementation of the universal HBV vaccination of infants, a nationwide survey sampling 61,702 participants across 30 provinces in mainland China found that the overall HBsAg positivity was 9.8% (range: 4.5–17.9%) [11]. In that survey, HBsAg positivity in male and female children aged ≤4 years was 10.7 and 8.5%, respectively; for male and female children aged 5–14 years the figures were 12.4 and 8.9%, respectively. Peak HBsAg positivity occurred in both males and females aged 10–14 years, but similar HBsAg-positive percentages remained until 50–59 years of age. In 2006, another national survey of HBsAg seroprevalence was conducted among individuals aged 1–59 years to update HBV epidemiology in China. The 2006 study found that HBsAg positivity had fallen to 7.2% in the general population (sample size: 81,775). Although 2.42% positivity was found in children aged 5–14 years, only 1.0% of children aged ≤4 years were HBsAg positive [4]. The latest national survey of hepatitis B virus infection conducted in 2014 (sample size: 31,713) only covered people aged 1–29 years. HBsAg prevalence among participants aged 1–4, 5–14, and 15–29 years was 0.3, 0.9, and 4.4%, respectively [3].

Sample sizes of the three prior national surveys were relatively small considering the population of mainland China is nearly 1.4 billion people. Moreover, few studies had larger sample sizes with wide range of age recently.

To update the current HBV infection in northeast China, we performed a serological survey of HBV infection in a large number of inpatients. This study also consisted of specialized disease cohorts. Admitted patients routinely received HBV-infection screening if high-risk factors for HBV infection were identified; if blood transfusions were received during surgeries, invasive procedures, dialysis, or pregnancy; or if iatrogenic infection and transmission were possible [12–14].

## Methods

### Study population

**Fig. 1:**
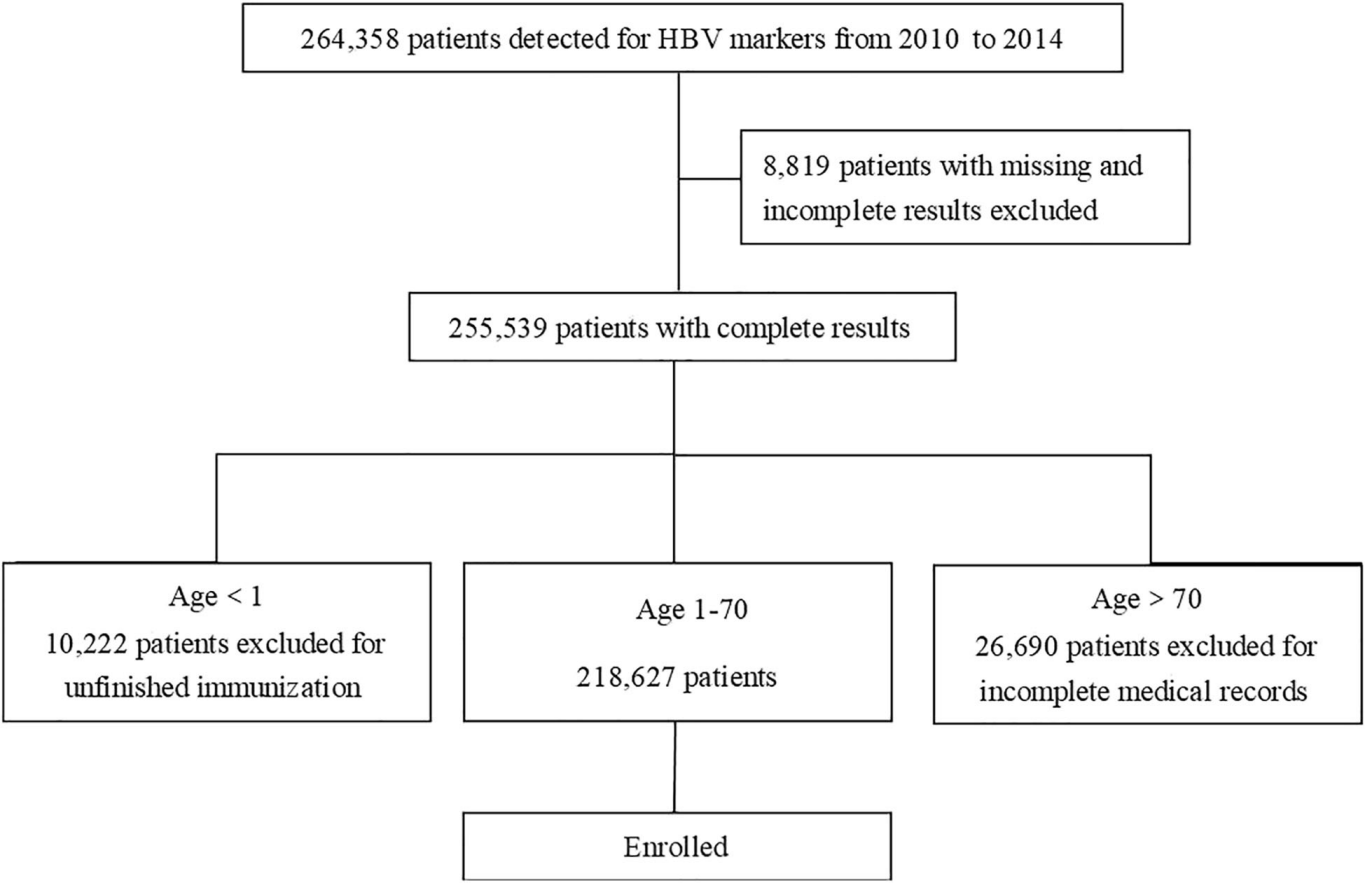
Patients selection flowchart

This cross-sectional study retrospectively reviewed the records of inpatients admitted to the First Hospital of Jilin University from January, 2010 through December, 2014. This hospital is the largest general hospital in Jilin Province with nearly 5500 inpatients beds. Patients come from not only the Changchun metropolitan region, but also from over 50 counties province-wide (over 27 million residents in 2014). A total of 694,826 admissions occurred during the study period. Patients receive HBV tests if indicated at high risk for HBV infection or before blood exposure including surgeries, invasive procedures, dialysis, and pregnancy. A total of 264,358 inpatients screened for HBV markers were initially included in the study (38.05% of all admissions). A small percentage—3.34% (8819 inpatients)—were excluded because of missing and incomplete data. Patients younger than 1 year or older than 70 years were excluded because of incomplete immunization and medical records. Finally, 218,627 patients were enrolled into this study (Fig. 1).

### Serological testing

Venous blood (3–5 ml) was collected in anticoagulated tubes from patients on the next morning after admission and separated within 24 h. All blood samples were screened using the same methods/techniques and the same valid reagents were provided by Abbott Laboratories. HBV serological markers including HBsAg, HBsAb, HBeAg, HBeAb, and HBcAb were quantitatively detected using chemiluminescence immunoassay (CLIA) in an Architect i2000 SR instrument (Abbott Laboratories, Lake Forest, IL, USA).

### Statistical analysis

The data for all subjects were collected and sorted using Microsoft Excel 2013 (Microsoft Corporation, New York, USA) software; SPSS v21.0 (IBM Analytics, New York, USA) software was used for the statistical analysis. Gender ratio and positivity rate for HBV markers in different age groups are expressed as percentages. The Chi-squared test (χ^2^) was used to compare the significances in differences between groups. *P* values < 0.05 were considered statistically significant.

## Results

### Characteristics of the study population (Table 1)

A total of 218,627 patients were finally included in this study. Aged ranged from 1 to 70 years (mean age, 43.38 ± 18.24 years). Patients were divided into 10-year age groups: 1–10, 11–20, 21–30, 31–40, 41–50, 51–60, and 61–70 years; 52.05% (*n* = 113,789) of the subjects were male and 47.95% (*n* = 104,838) were female.

**Table 1:** Positivity rate for five HBV markers in different groups

Variables		Total, n	HBsAg, %	HBsAb, %	HBeAg, %	HBeAb, %	HBcAb, %
Gender	Male	113,789	8.94	46.58	2.98	16.46	28.27
	Female	104,838	5.80	47.21	1.59	16.18	24.93
Age group	1–10	19,489	0.47	66.27	0.30	2.27	4.45
	11–20	9846	2.35	60.87	1.52	3.04	6.10
	21–30	20,685	6.74	53.48	3.28	11.95	19.59
	31–40	27,531	9.19	47.59	3.34	17.59	27.57
	41–50	48,475	10.05	41.35	3.17	19.42	30.57
	51–60	54,947	8.85	41.83	2.30	20.17	32.58
	61–70	37,654	6.04	43.54	1.19	18.97	33.12
Department	Cardiovascular	19,770	4.39	45.32	0.80	16.34	23.34
	Dermatology	1644	4.14	54.38	0.91	13.14	26.34
	Emergency	1981	12.17	42.66	4.49	21.76	47.40
	Endocrinology	988	12.96	45.65	3.74	24.09	47.27
	ENT	11,403	3.33	52.38	0.89	8.55	11.14
	Gastroenterology	2859	10.11	45.58	2.62	24.55	48.16
	Gynecology	3827	7.50	46.51	3.27	13.30	20.22
	Hepatology	9865	42.35	29.56	20.24	35.76	70.69
	ICU	702	10.40	48.01	3.28	24.22	48.86
	Infectious Diseases	3686	8.19	46.66	2.71	20.24	41.70
	Neurology	9417	5.72	42.36	1.17	17.50	26.72
	Obstetrics	12,124	5.22	47.77	1.28	15.25	20.24
	Oncology	6693	8.96	46.87	2.23	22.62	46.48
	Ophthalmology	2734	5.08	44.70	1.10	12.84	17.70
	Others*	3421	6.64	51.39	1.37	19.29	32.62
	Pediatrics	13,003	0.57	70.81	0.37	3.75	7.14
	Respiration	5253	7.25	45.84	1.87	21.19	44.87
	Rheumatology	3139	4.24	48.96	0.80	17.36	37.11
	Surgery	102,791	6.30	45.54	1.54	15.76	23.45
	Nephrology	3327	7.27	43.76	2.86	17.40	40.28
	Total	218,627	7.43	46.88	2.31	16.33	26.67

The positivity rate for HBV markers is presented as a percentage

There were significant differences in HBsAg, HBsAb, HBeAg and HBcAb positivity between genders. No significant differences between genders were found between genders in positivity rate for HBeAb

*VIP wards, Rehabilitation Department, Department of Traditional Chinese Medicine

### Distribution of HBV makers in different groups (Table 1)

Overall, 16,254 subjects (7.43%) were HBsAg positive. Males had a significantly higher HBsAg positivity than females (*P* < 0.001). HBsAg positivity varied significantly among different age groups. The highest rate was found in the 41–50 year age group (10.05%), and the lowest was in the under 10 years age group (0.47%). HBsAg positivity was the highest among patients with liver diseases; 6.2% of women of childbearing age(15–49 years) were HBsAg positive.

Nearly half of the enrolled patients were HBsAb positive. The total HBcAb positivity was 26.67%.

### Combinations of HBV markers in different groups (Table 2)

The positivity for more than one HBV marker reflected different HBV-infection statuses; 33.50–44.69% of the studied population were negative for any of the combinations of HBV markers. HBeAg-positive HBV infection accounted for 31.04% of the total HBsAg-positive population. Most HBeAg-positive patients were in the 21–30 year age group. Peak HBeAb positivity was seen in the 41–50 year age group. A singular HBsAb positivity reflected HBV vaccination and the highest rate was detected in the 1–10 year age group.

**Table 2:** Positivity rate for different combinations of HBV markers among different groups

Combinations		n	HBsAg+ HBeAg+ HBcAb+	HBsAg+ HBeAb+ HBcAb+	HBsAb+	HBsAb+ HBcAb+	HBeAb+ HBcAb+	HBcAb+	HBsAb+ HBeAb+ HBcAb+	All negative	Other combinations
Gender	Male	113,789	2.64	5.50	31.53	6.18	2.15	2.55	8.49	40.13	0.83
	Female	104,838	1.45	3.95	31.74	5.05	1.83	2.03	10.25	43.27	0.42
Age group (years)	1–10	19,489	0.26	0.05	62.21	1.56	0.02	0.19	2.08	33.50	0.13
	11–20	9846	1.41	0.71	57.33	1.19	0.05	0.35	2.14	36.59	0.22
	21–30	20,685	3.14	3.18	41.53	3.66	0.58	0.63	7.98	38.80	0.49
	31–40	27,531	2.99	5.48	31.56	5.19	1.29	1.38	10.62	40.74	0.76
	41–50	48,475	2.81	6.39	24.63	5.76	2.04	2.14	10.67	44.69	0.89
	51–60	54,947	2.03	6.07	23.79	6.82	2.87	3.15	10.95	43.56	0.77
	61–70	37,654	1.02	4.57	24.14	8.45	3.49	4.48	10.69	42.66	0.48
Total		218,627	2.07	4.76	31.63	5.64	2.00	2.30	9.33	41.64	0.64

### Comparison of HBsAg positivity between males and females in different age groups (Table 3)

HBsAg positivity in males was 8.94 and 5.80% in females. Male patients aged 41–50 years had the highest HBsAg positivity (13.36%), while in females the highest HBsAg positivity (6.90%) occurred in those aged 51–60 years. No significant difference was found between genders in patients aged under 20 years old. Higher HBsAg positive percentages were found in males than females in each age group in patients aged over 20 years old.

**Table 3:** Gender differences in the HBsAg-positive rate among all age groups

Age group (years)	Male		Female		*χ*2	*P*-value
	HBsAg, n	HBsAg, %	HBsAg, n	HBsAg, %		
1–10	67	0.51	25	0.39	1.437	0.231
11–20	137	2.37	94	2.31	0.038	0.846
21–30	765	8.04	629	5.63	47.278	< 0.01
31–40	1589	12.47	940	6.36	306.344	< 0.01
41–50	3178	13.36	1694	6.86	566.363	< 0.01
51–60	3068	10.6	1793	6.90	232.288	< 0.01
61–70	1369	6.86	906	5.12	49.927	< 0.01

Significant difference between genders in patients aged over 20 years

### HBcAb positivity at different age groups who were HBsAg positive or negative (Fig. 2)

**Fig. 2:**
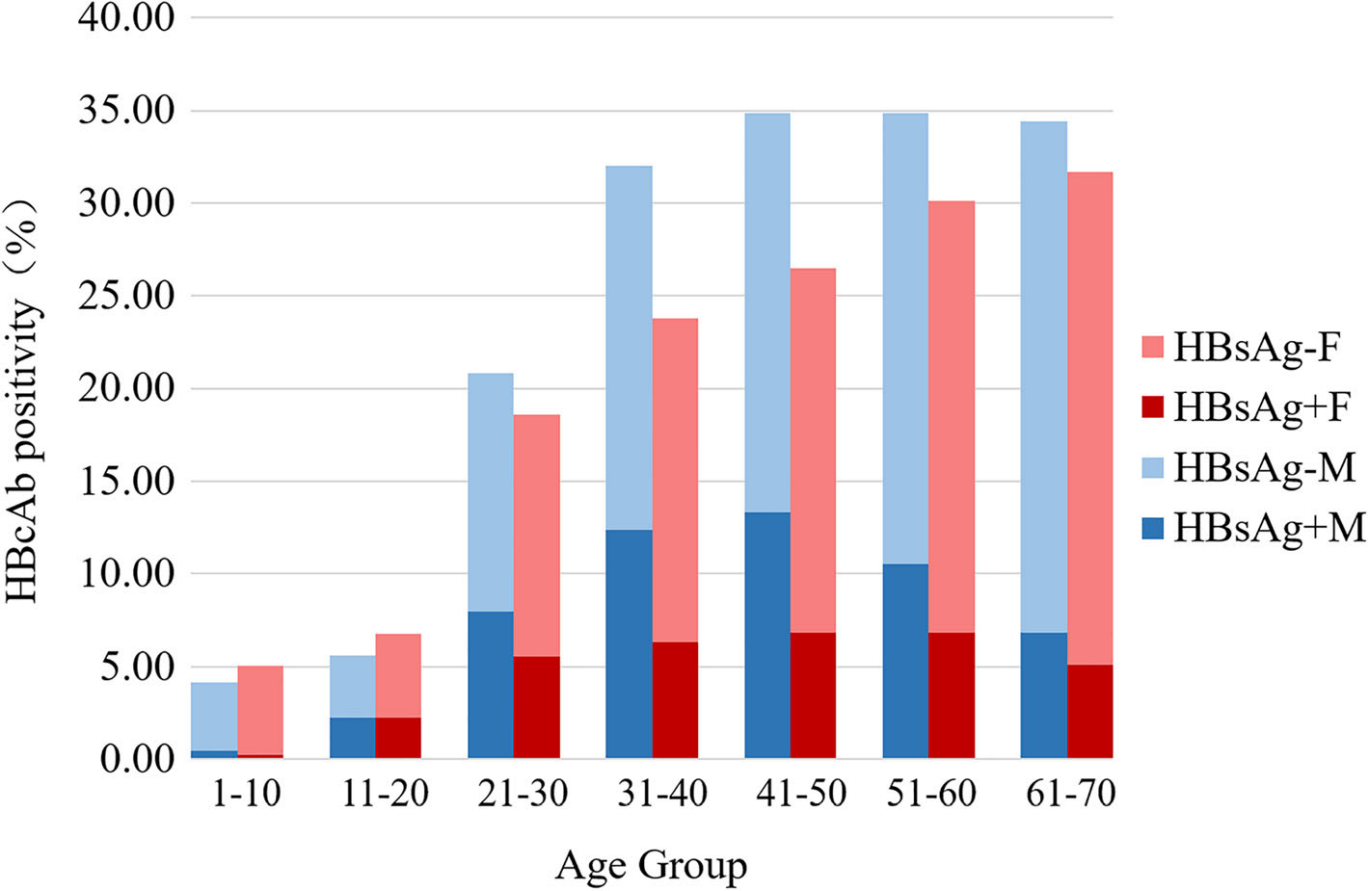
Distribution of HBcAb positivity by age groups M: Male, F: Female. Significant differences between male and female patients aged over 20 years.

Overall, 58,320 (26.67%) patients were HBcAb positive. Of those, 16,254 (27.87%) were HBsAg positive. Figure 2 shows the percentages of HBcAb positivity by HBsAg, gender, and age. The percentage of HBcAb positivity tended to increase with age. The HBcAb positivity in males plateaued after the age of 40 years, but kept increasing with age in females. For those aged over 20 years, HBcAb positivity in males was higher than in females. The percentages of HBsAg- and HBcAb+ inpatients also increased with age.

## Discussion

The number of participants enrolled in previous national surveys was 61,702 in 1992, 81,775 in 2006, and 31,713 in 2014, representing relatively small sample sizes for a national survey. The current study investigated HBV infection in a much larger sample of 218,627 hospitalized patients, and HBV serological markers were determined by CLIA, giving a higher sensitivity than enzyme-linked immunosorbent assay(ELISA) [15]. Results of this study reflect current HBV infection rates in northeast China. The main findings were (1) 6.2% of women at childbearing age were HBsAg positive; (2) HBsAg positivity in middle-aged male patients was 13.36%, which was the highest among all the age groups; and (3) 60% of patients aged under 20 years were only positive for HBsAb, which was the highest among all age group. Overall, nearly 40% of the study population lacked protection against HBV infection.

HBsAg positivity among participants aged 1–20 years was 1.1%. These patients were born after 1992 when hepatitis B vaccination of infants was first recommended. A dramatic decrease of HBV infection was seen in those patients born after 2002 when hepatitis B vaccination was formally integrated into routine immunization of infants. The impact of HBV vaccination on reducing HBV vertical transmission identified by this study was consistent with previous international studies [16–19]. In addition, the reduced HBsAg prevalence also reduced the incidence of liver cirrhosis and hepatocellular carcinoma [20]. The highest HBsAg positivity rate was detected in patients aged 41–50 years, reflecting the fact that they were likely infected before 1992 and continued to carry HBV infection until this study [21, 22]. Another factor was that those participants aged over 20 years tend to experience more risky behaviors, like unsafe injection and blood tranfusion, making them more vulnerable to HBV infection, compared with patients aged under 20 years.

The percentage of HBsAg positivity was higher in in the male age groups > 20 years than their female counterparts, and the difference between both genders increased with age. These results were consistent with the 2006 national survey and other studies [4, 23–25]. Androgen may inhibit both cellular and humoral immune responses. However, estrogen appears to only inhibit the cellular immune response while it may enhance the humoral immune response. In addition, progesterone promotes the transformation of cellular immunity to the humoral immune response [26, 27]. The sex hormones may contribute to the diverse between genders with age growing. Females were more likely to have HBsAg seroconversion. A notable finding among subjects aged under 20 years was that there was no significant difference in HBsAg positivity between males and females, as they had the same opportunity to access HBV vaccination independent of gender.

HBsAg positivity in women of childbearing age was 6.2%, which was much lower than the figure (11.8%) recorded from Fujian Province [28], which has a more developed economy than Jilin Province. This study conducted in 2013 explained that the high prevalence of HBsAg attributed to lack of consciousness from insufficient educational attainment and rural residency. HBsAg-positive pregnant women remain a significant challenge in blocking HBV vertical transmission as regular HBV vaccination schedule may fail to protect newborns from perinatal HBV infection.

Among patients aged under 20 years, HBsAg positivity increased with older age while HBsAb positivity decreased with older age. 60% of this population were HBsAb positive, reflecting successful HBV vaccination. This protective percentage resembled the result from Chengdu City [29]. Some factors, including low HBV vaccination coverage rate of 3 doses in rural areas, the quality of vaccines and cold chain operation for vaccine storage and transport, failed to make the HBsAb positivity higher in patients aged under 20 years. However the most important factor could be waning protection over time after the scheduled HBV vaccination. A previous study concluded that booster doses should be unnecessary for children after a full primary immunization owing to long lasting immune memory [30]; 41.64% of this population were negative for any HBV markers, suggesting they were neither exposed to HBV, nor vaccinated. If they were at high risk for HBV infection in the future, then HBV vaccination is highly recommended.

HBcAb positivity increased with age, independent of gender. As reported, HBcAb positivity increased with older age in younger age groups. However it then decreased in the oldest age groups [31], which is likely a consequence of spontaneous clearance, antiviral therapy, or increased mortality of cirrhosis and hepatocellular carcinoma [32]. HBsAg-negative/HBcAb-positive status is generally interpreted as having previous HBV exposure, but it could also indicate occult HBV infection [33], as defined as undetectable serum HBsAg accompanied by detectable HBV-DNA in serum and/or liver tissue [34]. A study from Italy found that more than 50% of HBcAb-positive subjects had detectable HBV DNA in their liver tissues [35]. In the current study, HBcAb positivity among HBsAg-negative patients was 19.29%, although we were unable to determine exactly how many of them had occult HBV infection. A recent study from Heilongjiang Province (a northeast neighbor to Jilin Province) [36] showed that nearly 1 out of 10 patients with negative HBsAg had occult HBV infection. Immunotherapy or chemotherapy may reactivate occult HBV infection and outcomes can be fatal. HBsAg-negative/anti-HBc-positive patients should therefore be closely monitored if their immunity is expected to be compromised by such a treatment or procedure.

Most surveys utilized ELISA for detecting HBV serological markers. However, CLIA was used in this study. Both ELISA and CLIA could equally detect HBV antigen and antibody titers. However, there is a significant difference in detection sensitivity between the two: CLIA is more sensitive (96.45%) compared to ELISA (77.3%) [37, 38] when serum HBsAg level is low [15].

Consistent with earlier studies [39, 40], nearly 14% of middle-aged male patients had HBV infection in this study and they were considered at high risk for advanced liver diseases and should be closely monitored. Many of these patients are unaware of their HBV-infection status, so a routine HBV marker test should be carried out in this group.

There are a few limitations in this study. First, the participants enrolled in this study are not representative of all inpatients or the general population since only patients who met the criteria for screening HBV infection were included. This population was based in a single hospital. The patients enrolled were not from random sampling. HBV infection prevalence detected in this inpatient population was expected to be higher while HBsAb positivity was lower comparing to the general population. Second, the patients in the study were only from Jilin Province, with no representation from outside this province. In future studies, we will include participants from throughout China to update HBV infection at a national level.

Our results highlight the critical impact of universal HBV vaccination of infants and young children on HBV infection prevalence in mainland China. Our findings reaffirm many previous studies, which showed significant decreases in HBsAg positivity mainly driven by the successful implementation of universal hepatitis B vaccination policy. Indeed, China has evolved from high to intermediate HBV endemicity. It is expected that endemicity will decrease further. Despite this remarkable achievement, China still faces an enormous challenge to manage nearly 100 million people with chronic HBV infection of who only 1% received standard antiviral treatment. One goal in managing HBV infection is to diagnose 90% of HBV infected cases by 2030 [41].

## Conclusions

HBsAg prevalence in young children (aged < 15 years) has declined from around 12% in 1992 to 0.47% in this study due to successful implantation of HBV vaccination of infants and young children during the past 22 years (until 2014) in China. However, more than 6.2% of women of childbearing age in this study were HBV infected and they may continue to vertically transit HBV infection if HBV vaccination fails to protect infants. In addition, China remains a country with nearly 100 million people that are chronically HBV infected.

Despite these challenges, we are committed to reducing HBV prevalence among children aged 5 years to 0.1% and to have 80% of eligible persons with chronic hepatitis B virus infection treated by 2030, which are goals set by the World Health Assembly (WHA) in 2016 [42].

## Abbreviations

CLIA: chemiluminescence immunoassay
ELISA: enzyme-linked immunosorbent assay
HBV: hepatitis B virus
WHA: World Health Assembly

## References

[1] Lu FM, Li T, Liu S, Zhuang H (2010). Epidemiology and prevention of hepatitis B virus infection in China. J Viral Hepat.

[2] Schweitzer A, Horn J, Mikolajczyk RT, Krause G, Ott JJ (2015). Estimations of worldwide prevalence of chronic hepatitis B virus infection: a systematic review of data published between 1965 and 2013. Lancet.

[3] Cui F, Shen L, Li L (2017). Prevention of chronic hepatitis B after 3 decades of escalating vaccination policy, China. Emerg Infect Dis.

[4] Liang X, Bi S, Yang W (2009). Epidemiological serosurvey of hepatitis B in China--declining HBV prevalence due to hepatitis B vaccination. Vaccine.

[5] Cui FQ, Wang XJ, Cao L, et al. Progress in hepatitis B prevention through universal infant vaccination--China, 1997-2006. Mmwr Morbidity & Mortality Weekly Report 2007. 56(18): 441–445. 17495790

[6] Liang X, Cui F, Hadler S (2013). Origins, design and implementation of the China GAVI project. Vaccine.

[7] Cui F, Liang X, Gong X (2013). Preventing hepatitis B though universal vaccination: reduction of inequalities through the GAVI China project. Vaccine.

[8] Hou J, Liu Z, Gu F (2011). Epidemiology and prevention of hepatitis B virus infection. Int J Med Sci.

[9] Wang AL, Qiao YP, Wang LH (2015). Integrated prevention of mother-to-child transmission for human immunodeficiency virus, syphilis and hepatitis B virus in China. Bull World Health Organ.

[10] Dai ZC Qi GM. Epidemiological investigation of viral hepatitis in China. Beijing. Beijing science and technology literature press. 1997: 19–20.

[11] Xia GL, Liu CB, Cao HL (1996). Prevalence of hepatitis B and C virus infections in the general Chinese population. Results from a nationwide cross-sectional seroepidemiologic study of hepatitis a, B, C, D, and E virus infections in China, 1992. Int Hepatol Commun.

[12] National Health and Family Planning Commission of the People's Republic of China. Viral Hepatitis Prevention and Control Plan of China (2017—2020). Chinese Journal of Viral Diseases. 2018. (1).

[13] Prevention of perinatal transmission of hepatitis B virus: prenatal screening of all pregnant women for hepatitis B surface antigen. MMWR Morb Mortal Wkly Rep. 1988. 37(22): 341–6, 351. 2967425

[14] Update: universal precautions for prevention of transmission of human immunodeficiency virus, hepatitis B virus, and other bloodborne pathogens in health-care settings. MMWR Morb Mortal Wkly Rep. 1988. 37(24): 377–82, 387–8. 2836717

[15] Yang L, Song LW, Fang LL (2016). Evaluation of a novel chemiluminescent microplate enzyme immunoassay for hepatitis B surface antigen detection. J Virol Methods.

[16] Ni YH, Huang LM, Chang MH (2007). Two decades of universal hepatitis B vaccination in Taiwan: impact and implication for future strategies. Gastroenterology.

[17] Gong J, Li RC, Yang JY (2003). Long-term efficacy of infant hepatitis B immunization program. Zhonghua Gan Zang Bing Za Zhi.

[18] Yonghao G, Jin X, Jun L (2015). An epidemiological serosurvey of hepatitis B virus shows evidence of declining prevalence due to hepatitis B vaccination in Central China. Int J Infect Dis.

[19] Zhu Q, Shao X, Chen S, et al. Epidemiological serosurvey of hepatitis B among children aged 1-14 in Guangdong Province, China. Int J Infect Dis. 2018. 10.1016/j.ijid.2018.01.027 29408358

[20] Chang MH, Chen CJ, Lai MS (1997). Universal hepatitis B vaccination in Taiwan and the incidence of hepatocellular carcinoma in children. Taiwan childhood hepatoma study group. N Engl J Med.

[21] Chen P, Yu C, Ruan B (2013). Prevalence of hepatitis B in insular regions of Southeast China: a community-based study. PLoS One.

[22] Wang CS, Chang TT, Yao WJ, Chou P (2002). Comparison of hepatitis B virus and hepatitis C virus prevalence and risk factors in a community-based study. American Journal of Tropical Medicine & Hygiene.

[23] Dutta S, Shivananda PG, Chatterjee A (1994). Prevalence of hepatitis B surface antigen and antibody among hospital admitted patients in Manipal. Indian J Public Health.

[24] Tripathi PC, Chakraverti TK, Khant NR. Seroprevalence of hepatitis B surface antigen and antibody to hepatitis C virus at a tertiary care Centre in Telangana. Lith Math J. 2015, 31(4):1.

[25] Gogos CA, Fouka KP, Nikiforidis G (2003). Prevalence of hepatitis B and C virus infection in the general population and selected groups in South-Western Greece. Eur J Epidemiol.

[26] Da SJA (1995). Sex hormones, glucocorticoids and autoimmunity: facts and hypotheses. Ann Rheum Dis.

[27] Wilder RL (1996). Adrenal and gonadal steroid hormone deficiency in the pathogenesis of rheumatoid arthritis. J Rheumatol Suppl.

[28] Zheng H, Cui FQ, Wang FZ (2018). The epidemiology of hepatitis B virus infection in women of reproductive age in highly endemic areas in China. J Viral Hepat.

[29] He F, Ma YJ, Zhou TY (2016). The serum anti-HBs level among children who received routine hepatitis B vaccination during infancy in Mianyang City, China: a cross-sectional study. Viral Immunol.

[30] Wu Q, Zhuang GH, Wang XL, Wang LR, Li N, Zhang M (2011). Antibody levels and immune memory 23 years after primary plasma-derived hepatitis B vaccination: results of a randomized placebo-controlled trial cohort from China where endemicity is high. Vaccine..

[31] Chu CM, Liaw YF (2007). HBsAg seroclearance in asymptomatic carriers of high endemic areas: appreciably high rates during a long-term follow-up. Hepatology..

[32] Chu CM, Liaw YF (2010). Hepatitis B surface antigen seroclearance during chronic HBV infection. Antivir Ther.

[33] Ba AF, Robertson PW, LePage AK, Jayamaha J, Baleriola C, Rawlinson WD (2013). The reliability of HBV core antibody in serological screening for hepatitis B virus. Pathology.

[34] Wong DK, Huang FY, Lai CL (2011). Occult hepatitis B infection and HBV replicative activity in patients with cryptogenic cause of hepatocellular carcinoma. Hepatology.

[35] Caviglia GP, Abate ML, Tandoi F (2018). Quantitation of HBV cccDNA in anti-HBc-positive liver donors by droplet digital PCR: a new tool to detect occult infection. J Hepatol.

[36] Fang Y, Shang QL, Liu JY (2009). Prevalence of occult hepatitis B virus infection among hepatopathy patients and healthy people in China. J Inf Secur.

[37] Madiyal M, Sagar S, Vishwanath S, Banerjee B, Eshwara VK, Chawla K (2016). Comparing assay performance of ELISA and Chemiluminescence immunoassay in detecting antibodies to hepatitis B surface antigen. Journal of Clinical & Diagnostic Research Jcdr.

[38] Liu C, Chen T, Lin J (2014). Evaluation of the performance of four methods for detection of hepatitis B surface antigen and their application for testing 116,455 specimens. J Virol Methods.

[39] Pereira LM, Martelli CM, Merchán-Hamann E (2009). Population-based multicentric survey of hepatitis B infection and risk factor differences among three regions in Brazil. Am J Trop Med Hyg.

[40] Fang JW, Lai CL, Chung HT, Wu PC, Lau JY (1994). Female children respond to recombinant hepatitis B vaccine with a higher titre than male. J Trop Pediatr.

[41] Organization WH. Global health sector strategy on viral hepatitis 2016–2021. Available from: https://www.who.int/hepatitis/strategy2016-2021/ghss-hep/en/.

[42] Global prevalence, treatment, and prevention of hepatitis B virus infection in 2016: a modelling study. Lancet Gastroenterol Hepatol. 2018. 3(6): 383–403. 10.1016/S2468-1253(18)30056-6 29599078

